# Invasive alien plant litter influences larval density, size and survival of *Culex* spp.

**DOI:** 10.1038/s41598-025-11556-z

**Published:** 2025-07-24

**Authors:** Tatenda Chiuya, Eric M. Fèvre, Joel Lutomiah, James Mutisya, Francis Mulwa, Betty Chelangat, Simon Muhoro, Richard Olubowa, Sandra Junglen, Christian Borgemeister

**Affiliations:** 1 Centre for Development Research (ZEF), University of Bonn, Genscherallee 3, 53113 Bonn, Germany; 2 International Livestock Research Institute (ILRI), Old Naivasha Road, PO Box 30709, Nairobi, 00100 Kenya; 3 Kenya Medical Research Institute (KEMRI), Centre for Virus Research, Nairobi, Kenya; 4 Institute of Infection, Veterinary and Ecological Sciences, University of Liverpool, Liverpool, L69 3BX UK; 5 Institute of Virology, Charité - Universitätsmedizin Berlin, University Berlin and Humboldt-University Berlin, Berlin, Germany

**Keywords:** Invasive alien plants, Culex, Life history traits, Physicochemical parameters, Mosquito-borne disease risk, Baringo County, Entomology, Invasive species, Viral vectors

## Abstract

Invasive alien plant (IAP) litter can alter the organic and inorganic content of mosquito breeding sites, influencing their life history traits. In Baringo County, Kenya, there is massive encroachment of *Prosopis juliflora* and *Parthenium hysterophorus* into crop and grazing lands. To mimic natural shedding, we set up experimental water containers in the field containing dried plant litter of either Parthenium, Prosopis or *Lantana camara*, which are invasive and *Acacia tortilis* which is native to the area. We measured water physicochemical parameters and counted mosquito larvae periodically for 6 weeks. After rearing, we determined survival rates and size of the emergent adults. All the plant treatments had significantly more larvae than the water-only control. Prosopis had the highest number of larvae which was significantly different from Acacia. Water conductivity, total dissolved solids and salinity were highest in Prosopis and Parthenium litter. For both *Culex pipiens* and *Culex vansomereni*, adults emerging from Prosopis and Parthenium litter were significantly larger in size compared to those from the other plants. Generally, for the two species, adults from IAPs survived longer than those from Acacia. Our study demonstrates the impact of IAPs on the life history traits of mosquitoes and how they can enhance their vectorial capacity. Mitigating the spread of these plants may reduce mosquito populations and risk of mosquito-borne disease.

**Supplementary Information:**

The online version contains supplementary material available at 10.1038/s41598-025-11556-z.

## Introduction

Mosquitoes are important vectors of diseases such as malaria, dengue, West Nile and Rift Valley fever which are a global health threat to humans and livestock^1^. These vectors breed in a wide range of natural and artificial aquatic habitats which can influence their development, distribution and abundance. Female mosquitoes are responsible for disease transmission as they are hematophagous while the males feed exclusively on plant nectar or juices^2^. Females also supplement their energy reserves with plant derived sugars influencing their fecundity, longevity and flight during host-seeking^2^. Terrestrial plants can influence life history traits of mosquitoes through plant feeding and the deposition of litter into their aquatic breeding habitats. Consequently, plant litter alters the organic and inorganic content of the breeding sites and also likely their pH, salinity and oxygen content all which have an impact on mosquito larval development^3^. With such dependence on plant nutrition and habitat, biodiversity shifts, due to deforestation, agriculture and invasive alien plants (IAPs) are likely to influence the occurrence of mosquitoes and therefore mosquito-borne diseases^4^. IAPs are particularly important as they can outcompete native species through production of abundant seeds, rapid germination, high growth and survival rate and allelopathy^5^. By displacing native plants, they provide a sugar source and habitat for mosquito breeding^6^.

The number of IAPs introduced into Africa has gradually increased over the years fueled by rising global trade and in some cases intentionally for reforestation in arid regions^6,7^. In Baringo County of the Kenyan Rift Valley, *Prosopis juliflora* (Family: Fabaceae; Prosopis onwards) is the major IAP while *Parthenium hysterophorus* (Family: Asteraceae; Parthenium onwards) and *Lantana camara* (Family: Verbenaceae; Lantana onwards) are also common^8,9^. Prosopis, was deliberately introduced in the 1980s into the area and promoted for its use in charcoal production to support local livelihoods and to improve soil carbon content^10^. It has rapidly spread, overtaking grasslands and suppressing native shrubs and trees^11^. Parthenium was accidentally introduced in Kenya in the 1970s^12^ and is now widely spread in areas along roads, waterways and on harvested agricultural plots. Lantana is mostly used for fencing^13^ with minimal presence in pastures and agricultural plots. One of the major indigenous tree species in the area is *Acacia* *tortilis* (Family: Fabaceae; Acacia onwards) which is often in direct spatial competition with Prosopis^8^. Note that the scientific names of Prosopis and Acacia (African species) have been recently updated to *Neltuma juliflora*  and *Vachellia tortilis* respectively but for consistency and clarity, the old names will be used in this study.

Litter input into aquatic bodies is dependent on the surrounding terrestrial vegetation and where IAPs dominate, debris from their leaves, fruits/seeds and flowers/pollen are also likely to dominate in these habitats altering their organic/inorganic content. Moreover, cues from decomposing IAPs may also influence the oviposition behavior of mosquitoes^14^. Previously, high water salinity and conductivity in breeding sites were associated with increased larval densities of *Anopheles* spp^15^. Generally, neutral/near-neutral pH is associated with higher mosquito species diversity compared to extremes (10 < pH < 4)^16^. Yet, some *Culex* spp. tolerate higher alkalinities, underlining that such effects are species-specific^16^. An increase in total dissolved solids (TDS), often used as a proxy for organic/inorganic content in water, is associated with higher larval densities in *Aedes* spp. breeding sites^17^.

Despite the widespread occurrence of IAPs, the influence of their litter on larval density in the field is poorly understood. The influence of plant litter on water physicochemical parameters and subsequently on larval density is also not clear. We therefore carried out a field experiment to investigate the effect of organic matter in water from three common IAPs in Baringo, i.e., Prosopis, Parthenium, Lantana and native Acacia, on larval density and life history traits (size and longevity) of container breeding mosquito species. We focused on *Culex* spp. the most abundant container breeding mosquito in Baringo which are also important primary vectors of West Nile fever and secondary vectors of Rift Valley fever. We also determined the influence of these litter infusions on the water physicochemical parameters in the breeding containers.

## Materials and methods

### Ethics declaration

Collection of plants, field and insectary experiments were carried out after approval by the International Livestock Research Institute (ILRI) Institutional Research Ethics Committee (refILRI-IREC2022-25) licensed by the National Commission for Science, Technology and Innovation (NACOSTI: License No: NACOSTI/P/22/19512) in Kenya.

### Study site

**Fig. 1:**
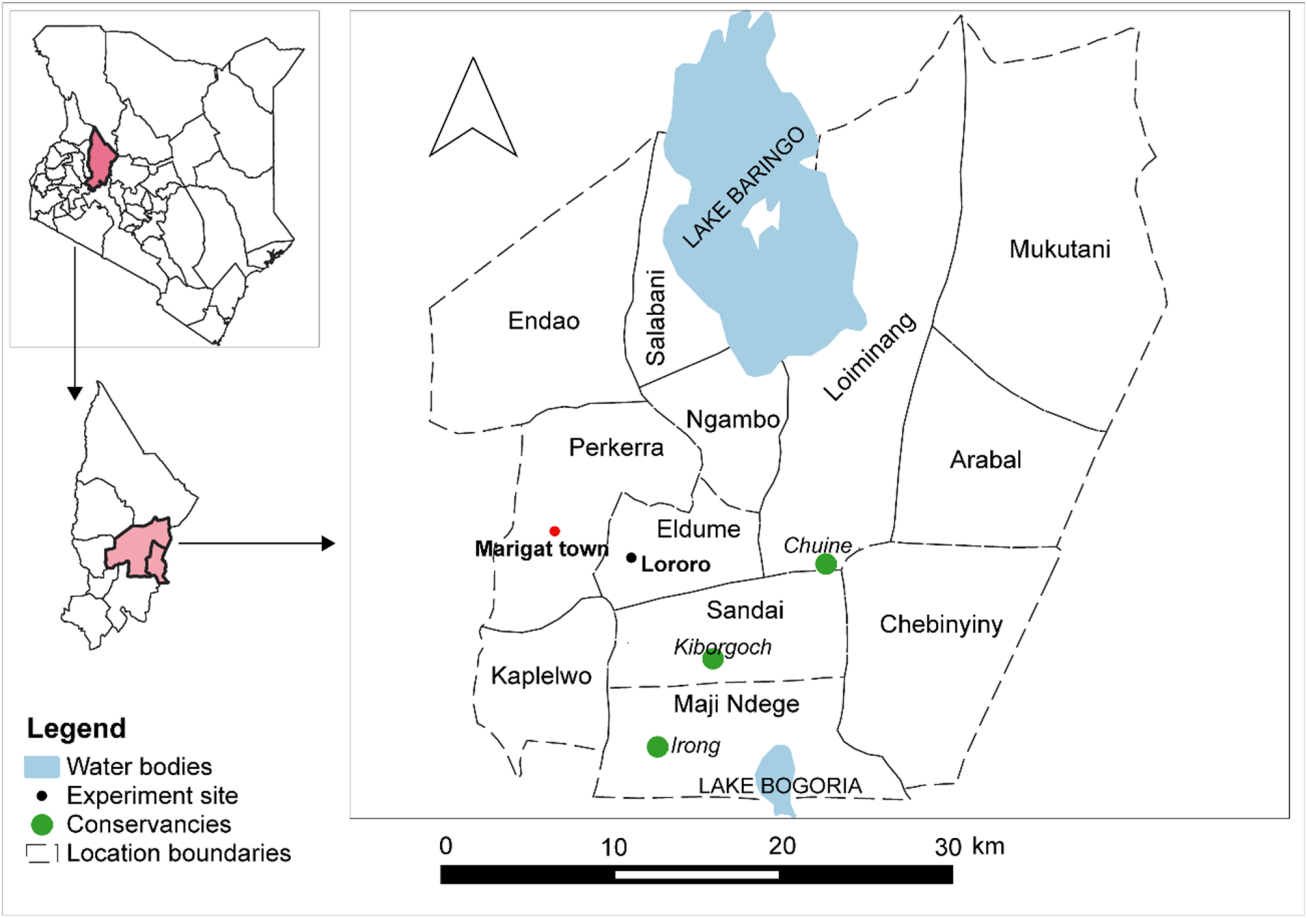
Map showing location of Baringo County, Kenya, Baringo South sub-County and Lororo village (homestead) where the experiment was set up.

The study was carried out in Lororo village of Baringo South sub-County, located about 10 km from Marigat town (Fig. 1). This area is semi-arid to arid with average annual precipitation of 650 mm and bimodal rainfall peaks in April and November^8^. Temperatures range from 30 to 35 °C with a monthly mean of 30 °C; however, highs of 37 °C are recorded in the hottest months of February and March^10^. The lowlands between the south shores of Lakes Baringo and Bogoria, specifically locations in and around Marigat, Ng’ambo, Salabani, Kiserian, Eldume, Ilng’arua, Loboi, Sandai and Kapkuikui are heavily infested with Prosopis (Fig. 1). The highest density is found in Ng’ambo where prosopis was initially planted in 1982^8,18^. Since then, it has invaded approximately 18,792 ha of land resulting in land use and land cover losses of native Acacia by 3452 ha (41%), grasslands by 2675 ha (37%) and mixed vegetation by 6215 ha (5%)^19^. In the same locations, 40% of 530 households sampled reported Parthenium invasions in their fields and pastures with about 65% ground coverage^9^. Based on our field observations, Lantana is also found in all these locations but is restricted to fences surrounding households and croplands where it is intertwined with Prosopis bushes. It is however not commonly found within grasslands or pastures.

The selected homestead in Lororo village was previously used during a mosquito trapping exercise. It is surrounded by Prosopis and scant Lantana bushes acting as a fence while some Prosopis trees also act as shade within the compound. In the vicinity of the homestead, maize and green grams are grown on several irrigated plots where Parthenium weeds are mostly found after the harvest.

### Plant litter Preparation

**Fig. 2:**
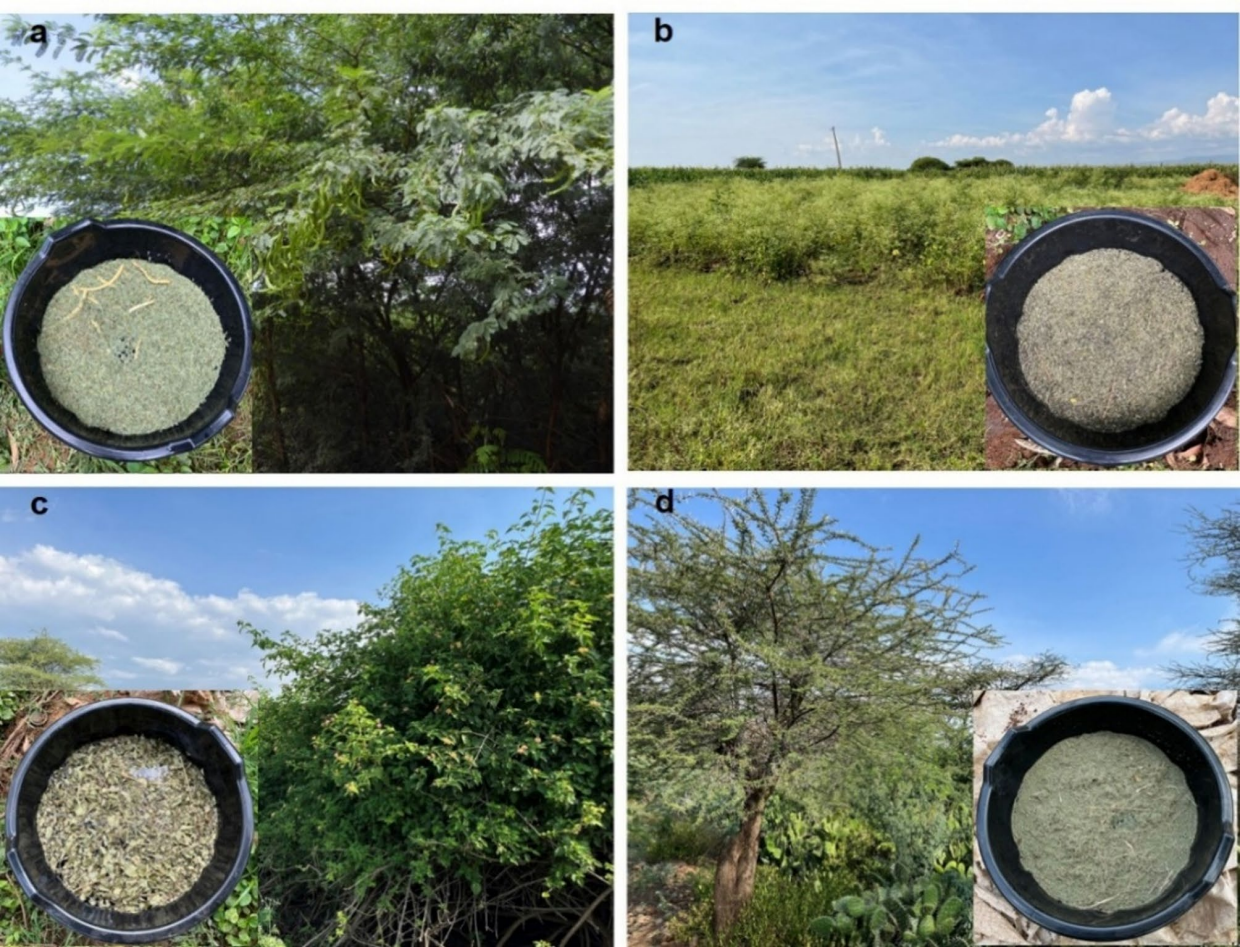
Image panel shows the plants that are found in the landscape of Marigat and Lororo village. Invasive plants, (**a**) Prosopis, (**b**) Parthenium, (**c**) Lantana, as well as the native plant (**d**) Acacia. Overlaid images in each panel show the containers with water and respective dried plant material.

Fresh plants were collected from the study area in March 2024 and air/sun-dried for 5 days before use in the experiment. All plants used in this study were collected with the assistance of Kenya Forestry Research Institute (KEFRI) research officers who confirmed the identity of each species in situ. For each plant, the leaves, upper stems, flowering parts, fruits/seeds and thorns were included. After air-drying, 50 g of each plant was weighed before use (Fig. 2).

**Fig. 3:**
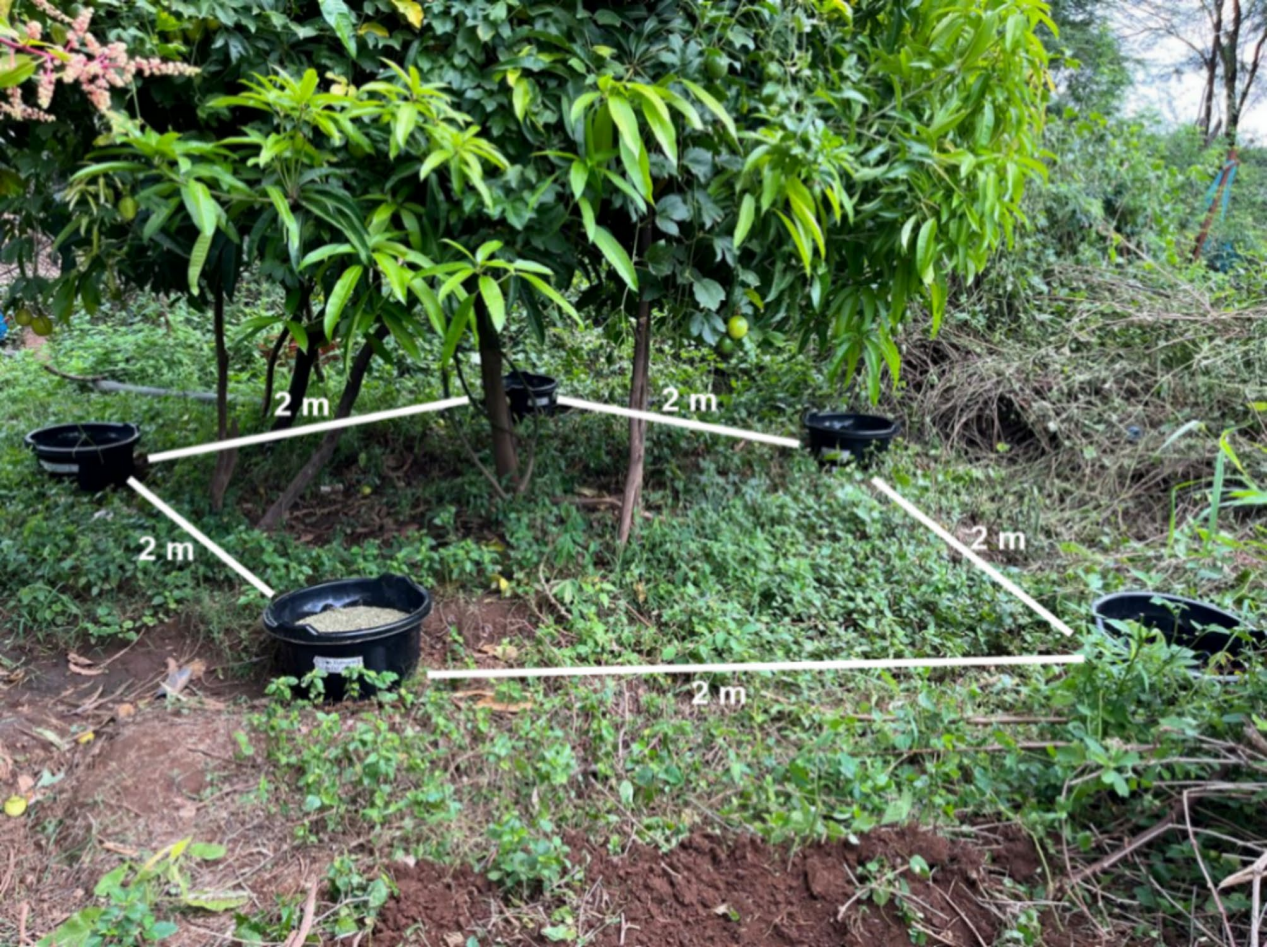
Arrangement of plant treatment containers in each replicate.

Plant litter was added to black containers containing 20 L of water. One container served as a control with water only (4 plant litter treatments + water-only control; Fig. 2). These were replicated 3 times at the homestead hence there was a total of 15 containers. The containers were arranged approximately 2 m apart to limit accidental oviposition among the containers. A regular sided pentagon arrangement allowed all the containers to evenly fit under the shaded areas provided by mango trees at the homestead. The 3 replicates themselves were also separated by 2 m (Fig. 3). All the containers were placed under the same type of tree (mango), allowing direct comparison of plant litter preference and minimize bias towards any specific plant litter. While volatile organic compounds from fruit trees may attract mosquitoes, this effect would have been uniform across all plant litter treatments and mosquitoes still had to choose based on cues from the experimental plants.

### Larval counting and measurement of water physicochemical parameters

The plant litter treatments were left in the field for 5 days to allow for larval colonization. After 5 days, larvae present in each container were counted and discarded and thereafter, every second day for 16 days in total. Container positions were rotated after every counting day such that each container occupied all the available positions in the set up. A calibrated generic 7-in-1 water quality meter (Milano, Europe) was used to measure physicochemical parameters of the water (pH, conductivity, TDS, salinity and temperature) in the plant litter treatments. Measurements were done on the day of setting up of the experiment and also on each day larval counting was done. To ascertain the consistency of our findings, 250 representative larvae were collected twice during the study period from each plant litter treatment and controls for rearing in the insectary. The results on mosquito size and survival analysis during the two sampling periods are reported separately in our results section. On the day of collection, larvae were transported in approximately 200 ml of plant litter treatment water to the insectary in whirl packs.

### Rearing and determination of adult body size

Larval rearing was done in a temperature- and humidity-controlled chamber. It was maintained at a constant temperature of 30 °C and approximately 80% relative humidity. The plant litter treatment water in the trays was replaced after 3 days with clean, chlorine-free water. Subsequent water changes were carried out every 2 days to provide optimal conditions for the larvae. Larvae were fed daily with TetraMin^®^ (Tetra^®^, Blacksburg, Virginia**)** fish food and once pupated, they were carefully transferred into netted cages labeled according to their respective plant litter treatment. Inside the cages, the adult mosquitoes were fed with a 10% glucose solution, using a cotton ball placed on the net with the solution being replaced every 2 days.

**Fig. 4:**
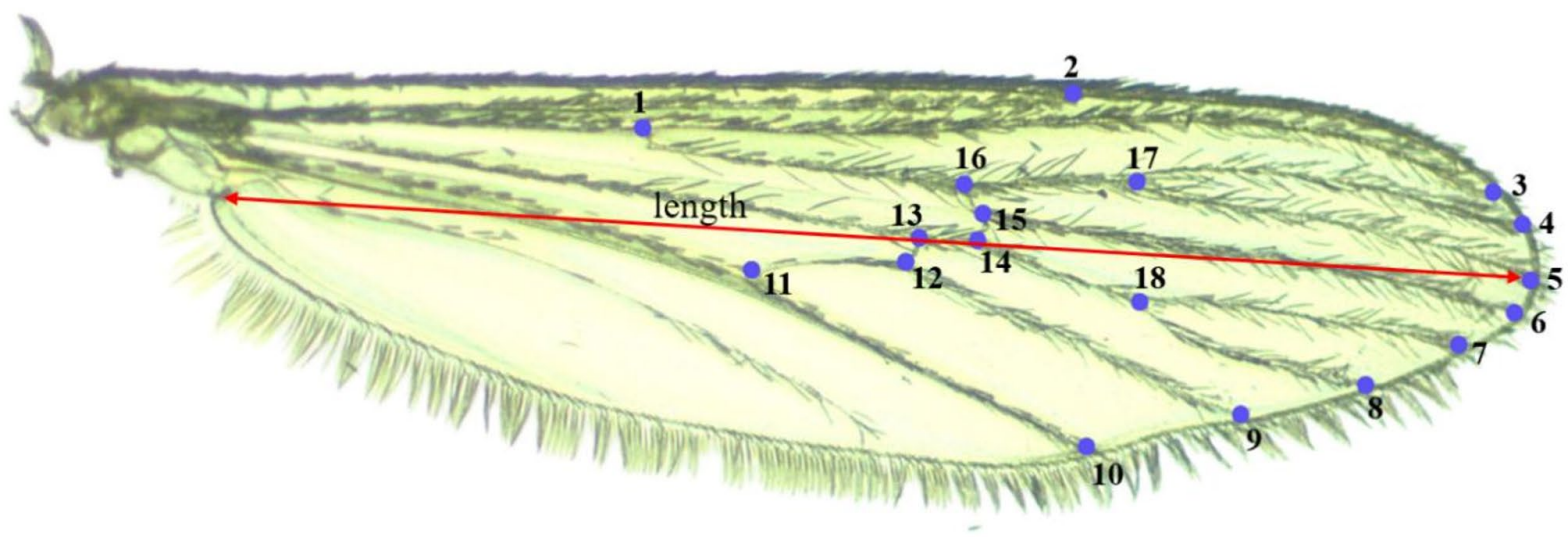
Landmarks on the dorsum of female *Culex pipiens* right wing that were used to calculate the centroid size.

After 4 days of glucose feeding, the glucose solution was replaced with water and mortality recorded daily. The dead mosquitoes were removed daily, identified using standard morphological identification keys^20,21^ and stored at −20 °C for later wing measurements. Ten random mosquitoes were selected per sex/species/treatment for body size determination. This was determined by measuring the wing centroid size which is a proxy for mosquito body size. The right wing was detached using fine forceps and its image captured using a Zeiss Axio-cam ERc 5 s digital camera (Göttingen, Germany) mounted on a stereomicroscope at X40 magnification. Using ImageJ software^22^ cartesian coordinates for 18 landmarks, were marked and used to calculate the centroid size of the wing (Fig. 4)^23^ using PAST software V.3.09^24^.

### Data analysis

All the data on physicochemical parameters of the plant litter treatment water and corresponding larval counts were stored in Microsoft Excel spreadsheets. The data were cleaned and descriptive statistics (means and standard deviation) calculated. Bar and line graphs were also constructed in Microsoft Excel. Means of the physicochemical parameters among plant litter treatments were compared using ANOVA with the Tukey’s HSD test as a *post-hoc* test. The mosquito count data was over-dispersed with few zeros hence a *negative binomial* generalized model was applied to test for differences in larval counts among the plant litter treatments. The same model was also used to determine the association between physicochemical parameters and larval counts. Due to expected high collinearity between TDS, conductivity and salinity, only pH and TDS were included in this model. Differences in mean centroid size of mosquitoes among the plant litter treatments were analyzed using a 2-way ANOVA. Kaplan-Meier and Log-Rank tests were used to compare survival curves and test whether the survival rates differed among the plant litter treatments. All statistical analyses were carried out in R (v4.3.1) software and significance level was inferred at *p* < 0.05.

## Results

### Effect of plant litter treatments on larval density and temporal variation

**Fig. 5:**
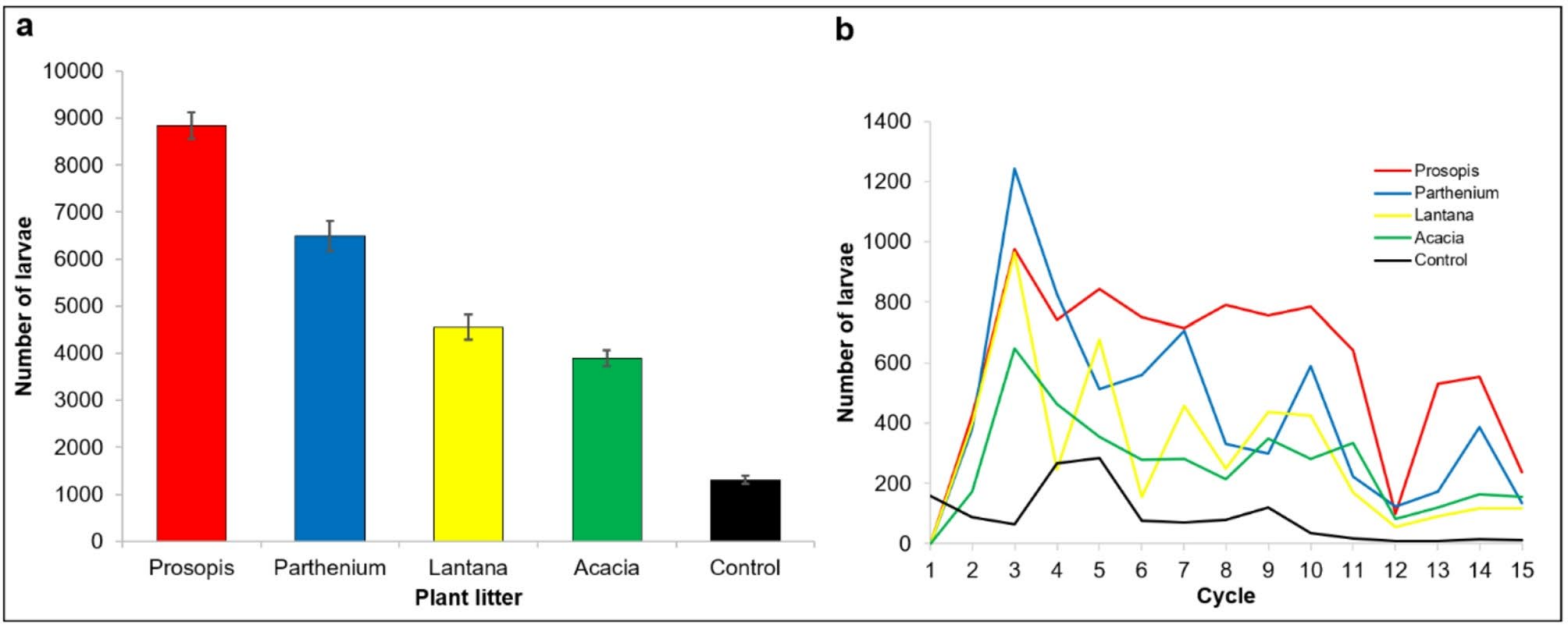
(**a**) Total mean mosquito larvae counts for each plant litter treatment and (**b**) the temporal variation over 6 weeks of study.

In the period between 23 June and 9 August 2024 we collected a total of 75,286 larvae from the 4 plant litter treatments and the control in three replicates. The mean larval counts for a period of 6 weeks are shown in Fig. 5a. The total mean count was 25,091 with the Prosopis litter treatment recording the highest mean count of 8,844 ± 284.5 followed by Parthenium (6,486 ± 321.4), Lantana (4,558 ± 260.9), Acacia (3,897 ± 161.2) and the control (1,306 ± 88.2). The temporal variation in the larval counts over the study period for each plant litter treatment and controls is shown in Fig. 5b. While Parthenium litter larval counts peaked in the first 5 counts and then fluctuated thereafter, the Prosopis counts subsequently remained higher than the other plants till cycle 12 when all treatments dipped. This dip in larval counts was due to a heavy downpour in the study area a few days preceding the 12th count.

There were twice as many larvae counted in the Prosopis litter treatment as compared to the Acacia treatment (OR = 2.34; CI = 1.26–4.35; *p* = 0.006) (Table 1). The difference in total larval counts between Acacia litter treatment and the other plants was, however, not significant. All the plant treatments had significantly more larvae counted than the water-only control. Incorporating temporal data as a categorical variable (divided into 3 phases: start, mid and end) in the model showed that overall, larval counts were at least 3 times higher in the first 2 weeks (first 5 counts) compared to the last 5 counts at the end of the study (Table 1) (OR = 3.27; CI = 1.99–5.38; *p* < 0.001).

**Table 1:** Differences in mosquito larval counts between plant litter treatments and time of counting.

Predictors	Mean larval count			
	Category	OR	95% CI	*p*-value
Plant litter				
	Prosopis	2.34	1.26–4.35	0.006
	Parthenium	1.53	0.83–2.85	0.170
	Lantana	1.01	0.54–1.89	0.965
	Control	0.25	0.13–0.47	< 0.001
	Acacia	ref	ref	
Time of count				
	mid	2.44	1.51–3.96	< 0.001
	start	3.27	1.99–5.38	< 0.001
	end	ref	ref	

### Effect of plant litter treatments on physicochemical parameters of water

**Fig. 6:**
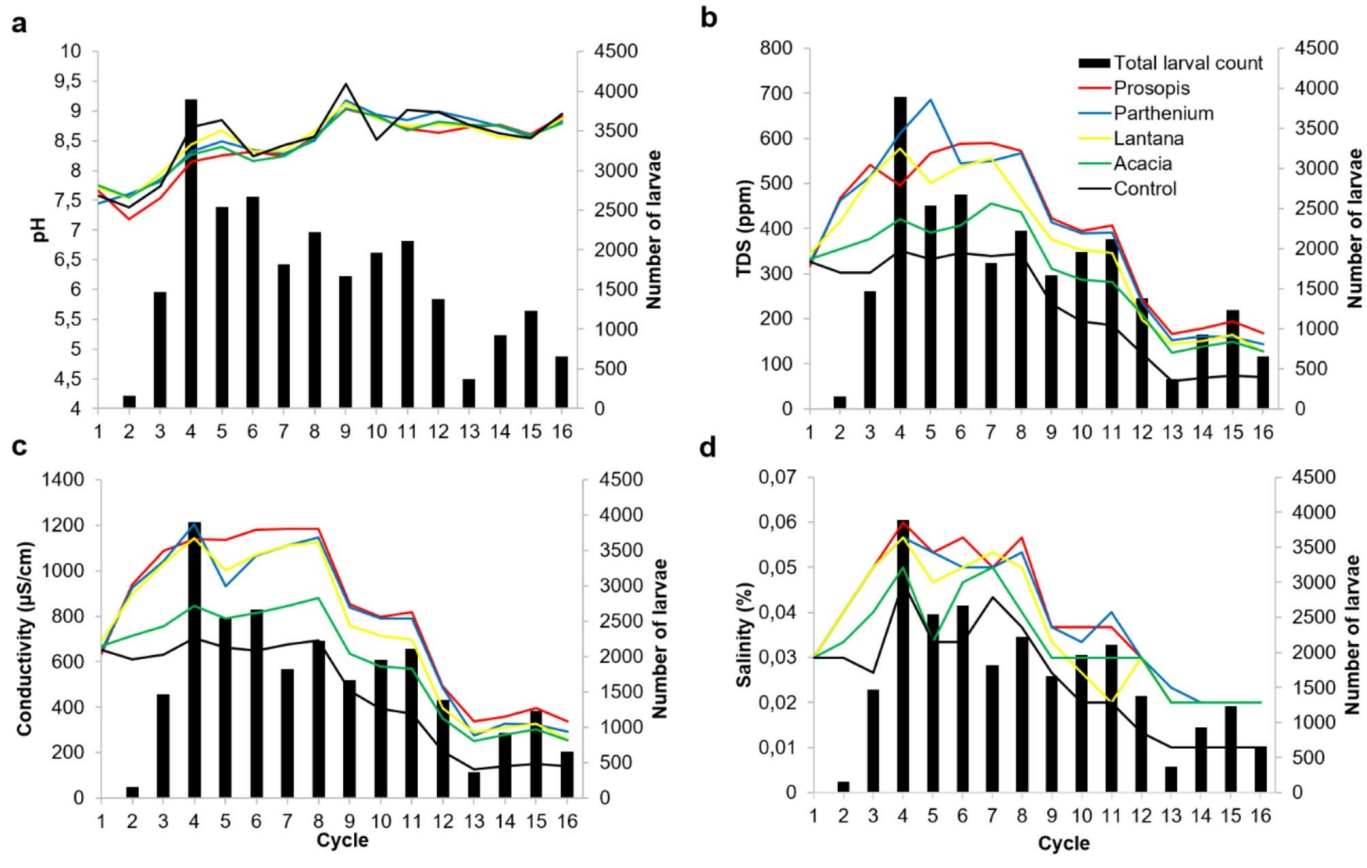
Temporal variation in mosquito larval counts as affected by (**a**) pH, (**b**) Total dissolved solids, (**c**) Conductivity and (**d**) Salinity of the water in each plant litter treatment and the control over 6 weeks.

The pH varied within a very narrow range over the study period for each of the plant litter treatments and did not seem to follow the trend in the total larval counts (Fig. 6a). On the other hand, the total larval counts closely followed the trend of TDS, conductivity and salinity (Fig. 6b, c, d). In the first month of the study these parameters were higher for Parthenium and Prosopis litter treatments compared to the other plants and the control (Fig. 6).

The pH in all the plant litter treatments was weakly basic while for all the other tested parameters the means were highest for Prosopis, Parthenium, Lantana and Acacia in decreasing order (Table 2).

**Table 2:** Distribution of mean levels of physicochemical parameters of the water in plant litter treatments.

Plant litter	Mean			
	pH	Conductivity (µS/cm)	Total dissolved solids (ppm)	Salinity (%)
Prosopis	8.4 ± 0.5	815.5 ± 342.9	399.8 ± 166.4	0.04 ± 0.01
Parthenium	8.6 ± 0.43	769.6 ± 340.1	399 ± 186.0	0.04 ± 0.01
Lantana	8.5 ± 0.4	742 ± 343.3	361.3 ± 166.1	0.04 ± 0.01
Acacia	8.5 ± 0.4	591.1 ± 241.2	298.3 ± 120.9	0.03 ± 0.01
Control	8.6 ± 0.5	441.1 ± 235.3	221.7 ± 117.0	0.02 ± 0.01

**Fig. 7:**
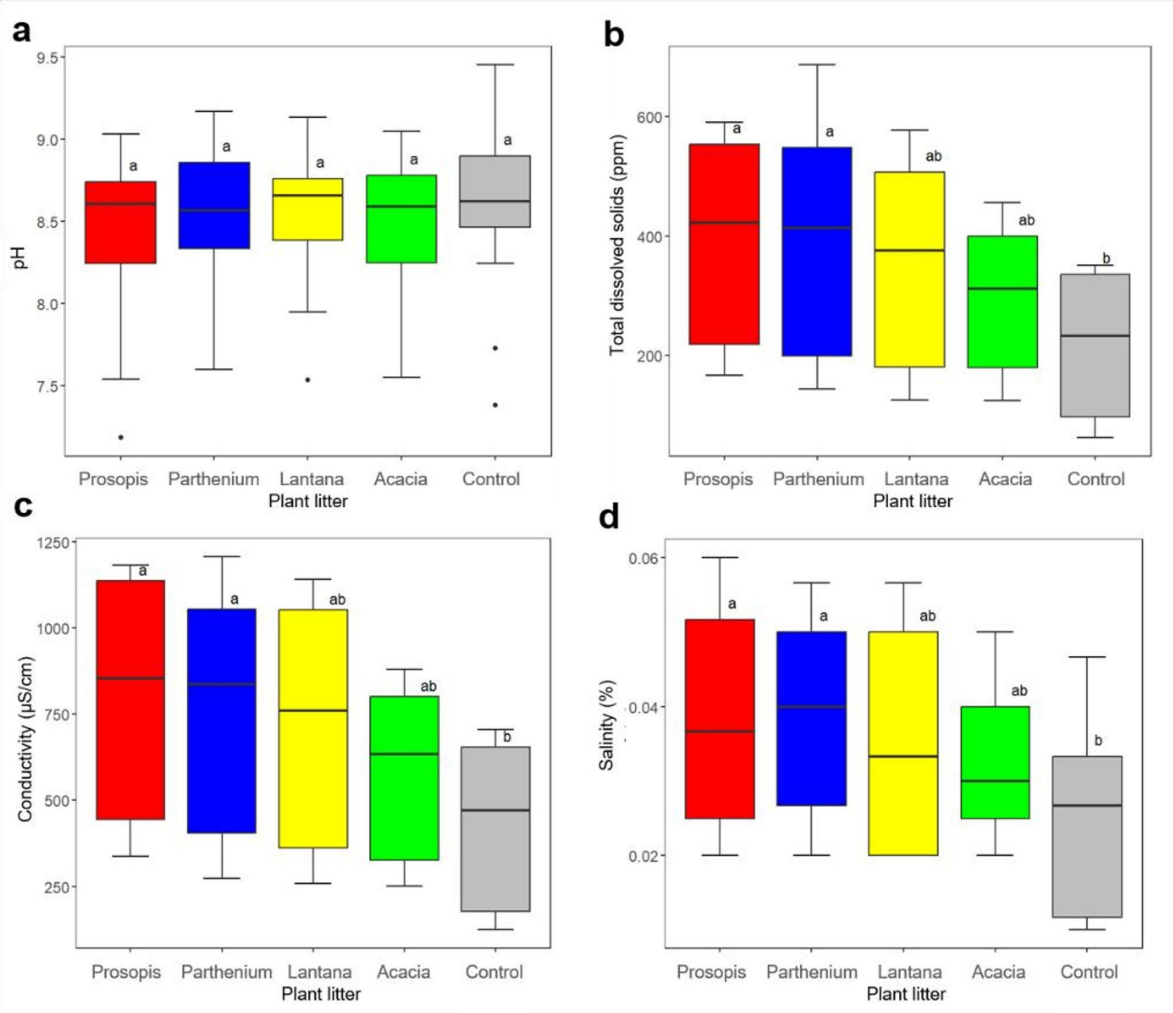
(**a**) pH, (**b**) Total dissolved solids, (**c**) Conductivity and (**d**) Salinity of plant litter treatments and the Control. Different letters among plant litter treatments indicate that differences were significant. Boxes show the interquartile range; the median is shown as a solid black line while the dots show outliers.

The ANOVA on the water physicochemical parameters revealed no significant differences in pH among the treatments and the control (Fig. 7a). Prosopis (F _(4,70)_ = 400; *p* = 0.018) and Parthenium (F _(4,70)_ = 399; *p* = 0.019) had significantly higher TDS when compared to the control (Fig. 7b). For the conductivity, Prosopis (F _(4,70)_ = 816; *p* = 0.01) and Parthenium (F _(4,70)_ = 770; *p* = 0.034) had significantly higher values compared to the control (Fig. 7c). Prosopis (F _(4,70)_ = 0.04; *p* = 0.03) and Parthenium (F _(4,70)_ = 0.04; *p* = 0.04) also had significantly higher salinity compared to the control (Fig. 7d). However, in the 3 parameters, the other plant treatments were not significantly different from each other and the control.

Scatter plots showed that conductivity, TDS and salinity were highly correlated; however, TDS is more likely to influence the abundance of mosquito larvae than the other 2 parameters. Yet, TDS was not significantly associated with larva counts (OR = 1.00; 95% CI = 1.00–1.01; *p* < 0.001).

### Effect of plant litter treatments on mosquito body size

The most abundant mosquito species were selected for survival analysis and body size determination. For the two larval sampling periods, the most abundant species emerging from all the plant litter treatments were *Culex vansomereni* and *Culex pipiens*. The diversity of adults that emerged from the litter treatments is shown in Supplementary Table 1.

**Fig. 8:**
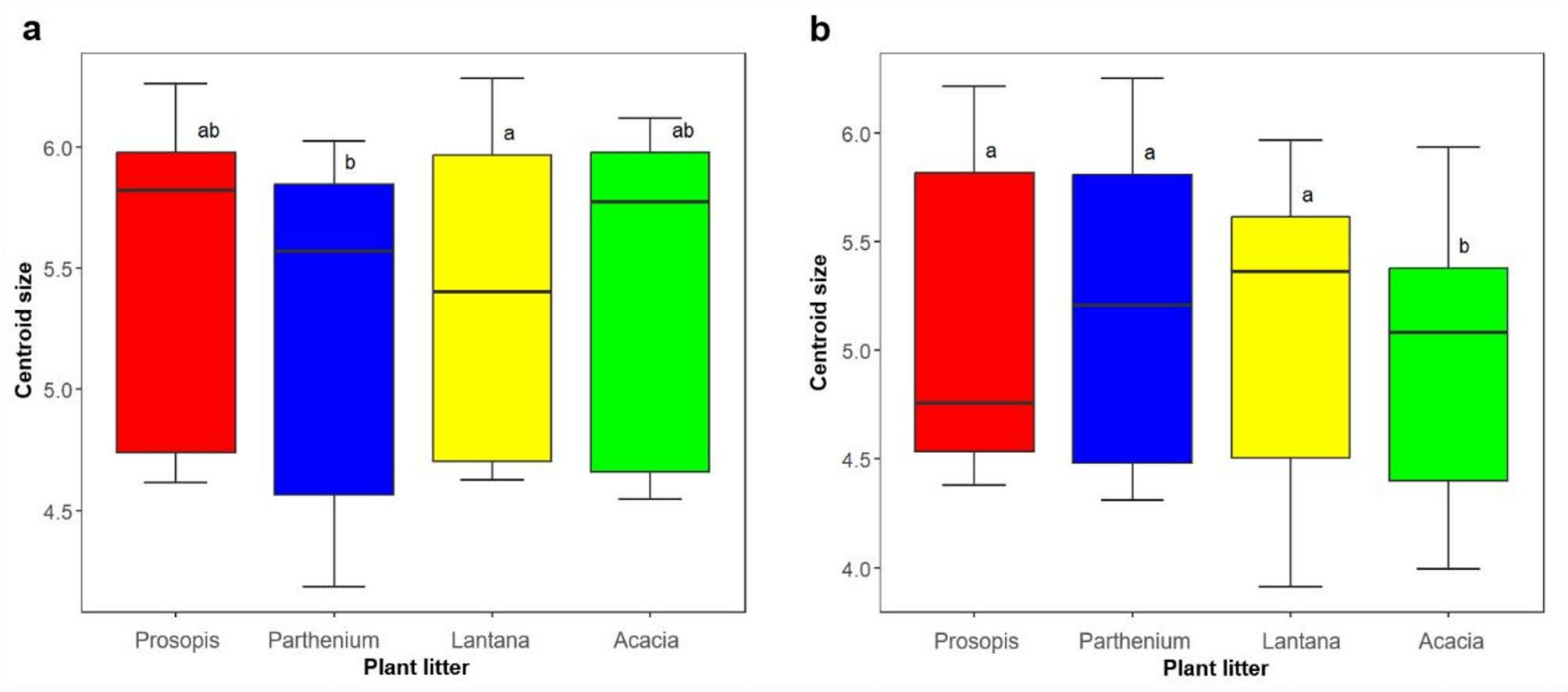
Size of *Culex pipiens* adults from different plant litter treatments in (**a**) the first larval sampling period and (**b**) second sampling period. Significant differences are shown by different letters. Boxes show the interquartile range and the median is shown as a solid black line.

In the first larval sampling period and rearing, plant litter treatment had a significant main effect on adult mosquito size (F _(3,45)_ = 4.62, *p* = 0.007) of *Cx. pipiens*. Adults emerging from Lantana litter treatment were significantly larger (mean = 5.37 ± 0.7; *p* = 0.007) compared to those from Parthenium (mean = 5.22 ± 0.7) (Fig. 8a). However, those emerging from the other plant litter treatments were not significantly different in size (Fig. 8a). In the second sampling period, plant litter treatment also had a significant main effect on mosquito size (F _(3,64)_ = 6.93, *p* < 0.001) of *Cx. pipiens*. Adults emerging from Prosopis (mean = 5.14 ± 0.7; *p* < 0.001), Parthenium (mean = 5.19 ± 0.7; *p* = 0.003) and Lantana (mean = 5.14 ± 0.6; *p* = 0.001) litter treatments were significantly larger compared to those from Acacia (mean = 4.97 ± 0.7). *Cx. pipiens* adults emerging from the other plant litter were not significantly different in size on pairwise comparison (Fig. 8b).

For *Cx. vansomereni* in the first larval sampling, there was a significant interaction between plant litter treatment and sex (F _(3, 48)_ = 45.15, *p* < 0.001), indicating that the effect of plant litter treatment on adult mosquito size differed between males and females. Female *Cx. vansomereni* from Prosopis (mean = 6.16 ± 0.2) were significantly larger than females from all the other plant litter treatments, while Parthenium (mean = 5.72 ± 0.2; *p* < 0.001) and Lantana (mean = 5.86 ± 0.2; *p* < 0.001) litter treatments resulted in significantly larger mosquitoes than Acacia litter (mean = 4.62 ± 0.2). Male *Cx. vansomereni* adults emerging from Prosopis litter (mean = 4.77 ± 0.1) treatments were larger than those in all the other litter treatments. However, this difference was not significant with Acacia litter (mean = 4.71 ± 0.1). Males from Acacia were significantly larger (mean = 4.71 ± 0.1; *p* < 0.001) than the ones from Parthenium (mean = 4.30 ± 0.1). There were no significant differences in mosquito size among the other pairwise comparisons.

In the second sampling period, there was also a significant interaction between plant litter treatment and sex, for *Cx. vansomereni* size (F _(3, 44)_ = 4.14, *p* = 0.01). There was no significant difference in size among female *Cx. vansomereni* emerging from all the plant litter treatments. However, those from Prosopis (mean = 5.83 ± 0.3) were the largest while the ones from Acacia (mean = 5.66 ± 0.3) were the smallest. Male *Cx. vansomereni* emerging from Lantana litter (mean = 4.49 ± 0.3) were larger than those from Acacia (mean = 3.98 ± 0.3); (*p* = 0.002) and Prosopis (mean = 3.95 ± 0.2); (*p* < 0.001) while the ones from Parthenium litter were larger (mean = 4.33 ± 0.3; *p* = 0.02) than the ones from Prosopis (Mean = 3.95 ± 0.2).

### Effect of plant litter treatments on mosquito survival

**Fig. 9:**
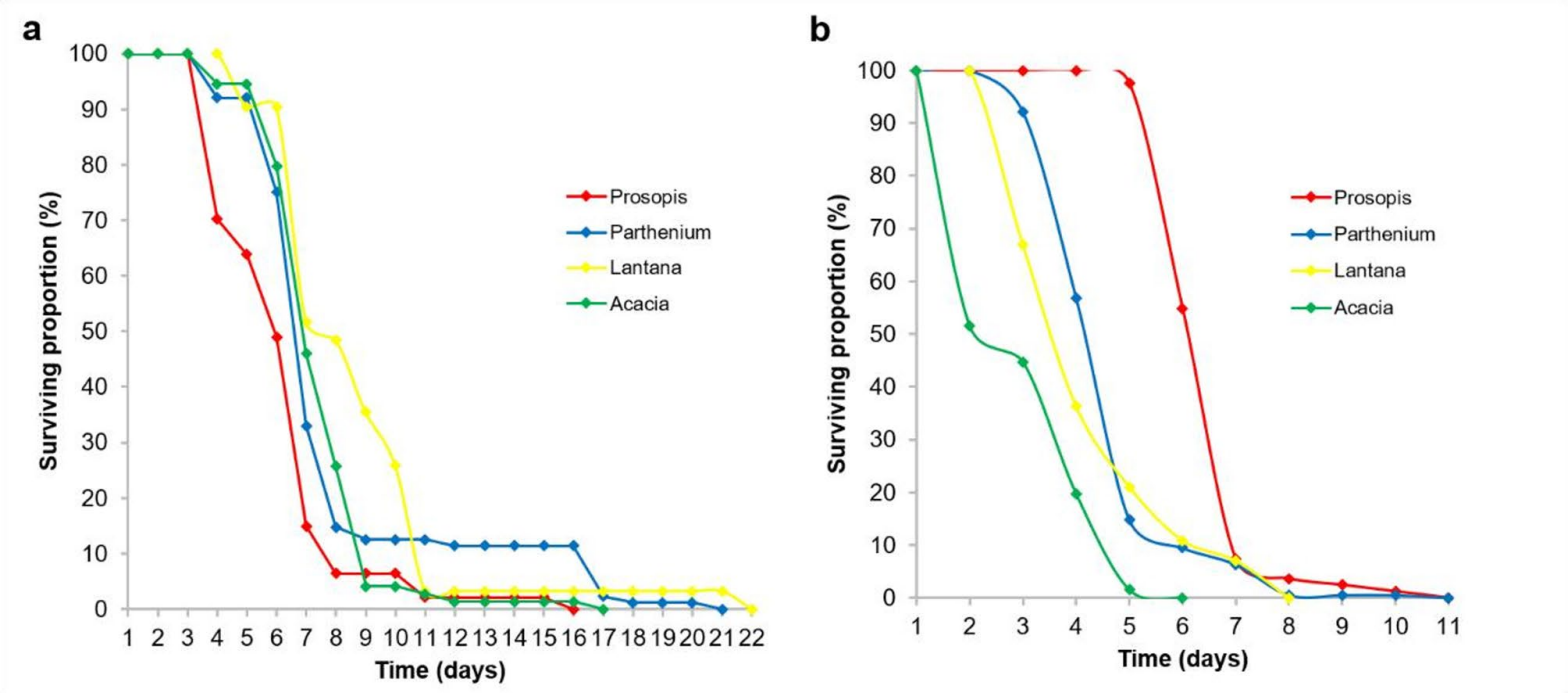
Survival probabilities over time of *Culex pipiens* adults emerging from different plant litter treatments based on a Kaplan-Meir survival estimator model in the (**a**) first and (**b**) second sampling period.

In the first larval sampling period, *Cx. pipiens* adults from Lantana litter survived significantly longer than those from Acacia (*p* = 0.042) and Prosopis litter (*p* < 0.001) while those from Acacia survived significantly longer than those from Prosopis litter (*p* = 0.001). The ones originating from Parthenium litter also survived significantly longer than those from Prosopis (*p* = 0.001). (Fig. 9a). In the second sampling, considering survival indices and curves, *Cx. pipiens* adults emerging from invasive plant litter survived significantly longer (Prosopis = *p* < 0.001; Parthenium = *p* < 0.001; Lantana = *p* < 0.001) than those emerging from native Acacia litter treatments (Fig. 9b).

**Fig. 10:**
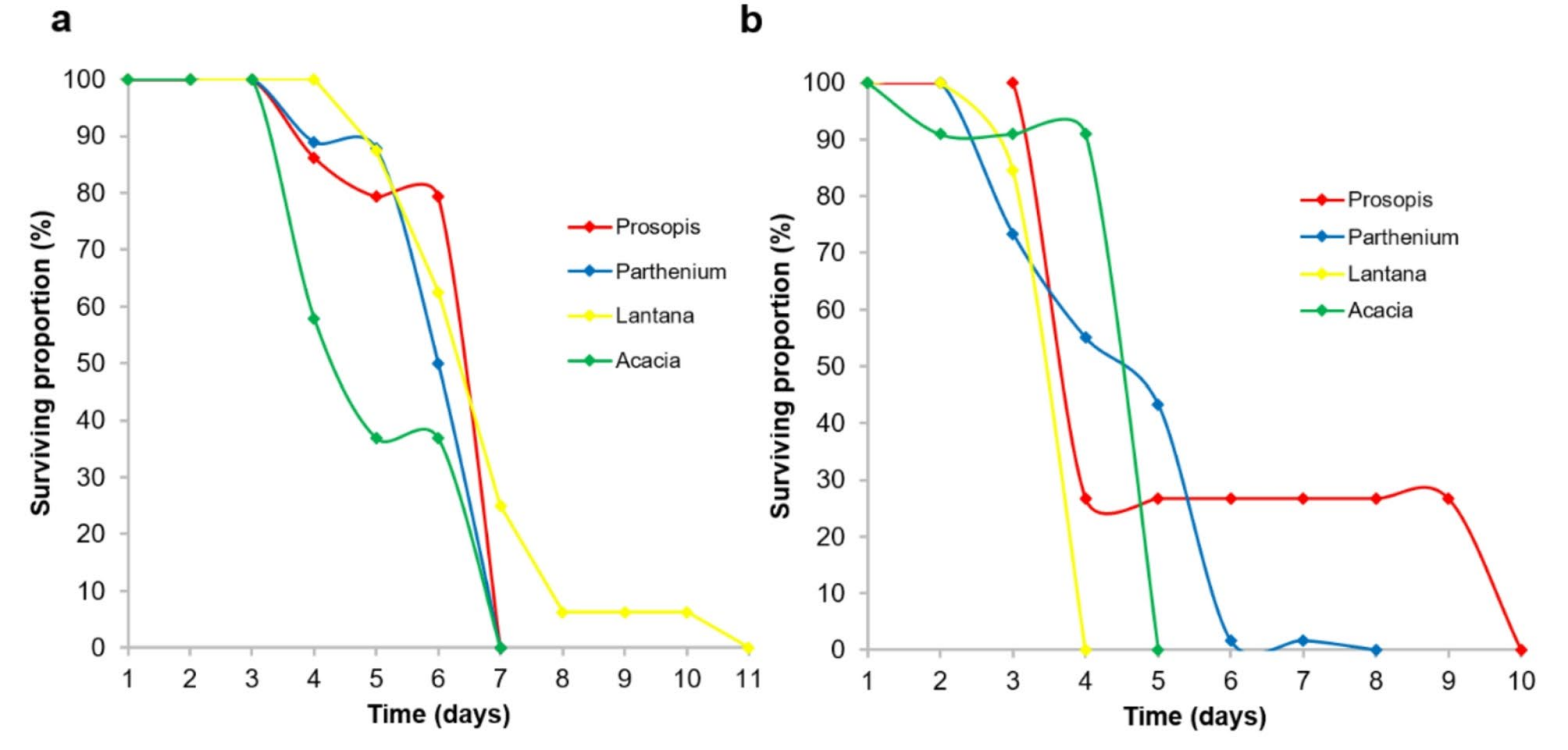
Survival probabilities over time of *Culex vansomereni* adults emerging from different plant litter treatments based on a Kaplan-Meir survival estimator model in the (**a**) first sampling period and (**b**) second sampling period.

For *Cx. vansomereni*, in the first larval sampling, emerging adults from Prosopis litter survived significantly longer than those from Acacia (*p* = 0.02). The other pairwise comparisons were not significantly different (Fig. 10a). In the second sampling, *Cx. vansomereni* adults emerging from Prosopis (*p* = 0.002), Parthenium (*p* < 0.001) and Acacia (*p* < 0.001) survived significantly longer than those from Lantana. (Fig. 10b).

## Discussion

Litter from terrestrial vegetation is a major determinant of the organic composition of nearby aquatic bodies that may serve as mosquito breeding habitats. However, little is known about how this input from certain IAPs affects the immature stages of mosquitoes that breed in these habitats. Our results show that water containing organic matter from Prosopis litter and Parthenium were the most attractive oviposition sites to gravid mosquitoes compared to native Acacia and water-only controls. The former two also sustained higher water physicochemical parameters and in general resulted in larger *Cx. pipiens* and *Cx. vansomereni* mosquitoes than the litter from other plants. The effect of Prosopis and Parthenium on mosquito longevity was not consistently observed between the two species in the two larval sampling periods. However, in general, mosquitoes from IAPs survived longer than those from the native Acacia litter. These findings illustrate how the displacement of native plants by IAPs can influence *Culex* populations at multiple life cycle stages, their survival and fitness and, subsequently, transmission of arboviruses.

Prosopis litter resulted in the highest number of *Culex* larvae in our experiment over the 6 weeks of study which could be attributed to its contribution to the organic composition of the habitat and rapid decomposition rate. IAPs decompose faster than native species leading to a greater attraction of gravid mosquitoes, especially *Cx. pipiens* that favor habitats with high organic content^25^. Aligning with our findings, a study in South Africa, found that IAPs such as Lantana and Guava, *Psidium guajava* (Myrtaceae), drove higher larval densities compared to native plant species^26^. Also, in concurrence on the effect of IAPs mosquito on breeding, in the USA, higher adult emergent rates of *Cx*. *pipiens* were associated with the invasive honeysuckle, *Lonicera maackii* (Caprifoliaceae), compared to native plants such as blackberry, *Rubus allegheniensis* (Rosaceae). This difference was attributed to the rapid decomposition rate and higher microbial populations in honeysuckle compared to the native plants^27^. Bacteria generate chemical cues that attract gravid mosquitoes and are also a source of food for mosquito larvae^28^. For example, Enterobacteriaceae associated with Parthenium root exudates were found to be responsible for cues that attracted gravid *Anopheles gambiae*^29^. With Prosopis and Parthenium invasions also happening in Ethiopia, Uganda, Eritrea, Tanzania, Madagascar, Mozambique, Zimbabwe, Mauritius and Eswatini, our findings highlight a broader ecological driven risk of mosquito-borne diseases in eastern and southern Africa^30–32^.

The difference in larval counts we found is likely to reflect oviposition preference based on chemical cues emanating from the decomposing plant litter. Even though not enumerated, a higher number of egg rafts were observed in Prosopis and Parthenium plant litter compared to Lantana and Acacia. However, differences in larva counts could be partly attributed to egg hatching and larval survival in the plant litter. The larvicidal effects of natural compounds from these plants have not been conclusively described, with methanol leaf extracts of Prosopis found to be larvicidal against *Cx*. *quinquefasciatus*^33^ while Parthenium showed the same effect on *Aedes aegypti* and *An. arabiensis* larvae^34^. Lantana has shown both repellency and larvicidal properties against adult *Ae. aegypti*^35^ and larvae of *An. culicifacies*^36^ respectively. In another study, acetone extracts of Acacia had a very strong larvicidal effects on *Cx. pipiens* and *Ae. aegypti*^37^.

Prosopis and Parthenium also had the highest physicochemical parameters such as TDS, salinity and conductivity compared to the other studied plants. TDS is an important parameter as it is likely to influence salinity/conductivity and represents the organic and inorganic components of the habitat. Ammonia and nitrate composition of the habitat has a greater influence in the breeding of *Ae. aegypti* larvae but less on *Culex* spp., alluding to the greater chemical tolerance of *Culex* spp^38,39^. In our study none of the physicochemical parameters were significant predictors of *Culex* spp. larval abundance/count. While this has been previously reported^15^, in our case it could be also attributed to the dilution effect of the heavy rains that fell in the last third of the experiment. Additionally, other factors other than decaying litter such as soil type and water quality may contribute to physicochemical parameters of natural aquatic habitats. These factors were absent in our experimental artificial containers.

While a clear trend on the effect of specific plant on mosquito survival was not evident, mosquitoes from IAPs in general survived longer than those from Acacia. These inconsistencies in the longevity of mosquitoes are expected because besides nutrition, both survival and body size are also amenable to other factors affecting larvae such as temperature and density^40^. Also, as mentioned before, the prevailing environmental conditions during the two sampling periods were slightly different. Nevertheless, this IAP driven increase in mosquito survival is crucial in disease transmission as mosquito longevity is the most important parameter of vectorial capacity^41^. Prosopis and Parthenium resulted in larger *Cx. pipiens* and *Cx. vansomereni* mosquitoes in comparison to the native Acacia, especially in females. This can be traced back to the higher organic content in Prosopis and Parthenium, and shows the carry-over effect of larval habitats to the adult stage^42^. The same study also showed that in addition to supporting higher emergence rates in *Cx. pipiens*, honeysuckle also resulted in larger mosquitoes. Larger mosquitoes emerge with more nutritional reserves at eclosion increasing their flight potential to contact hosts and thus their vectorial capacity. Also, for females their initial blood-meals are immediately utilized in the reproductive cycle compared to smaller females that have to feed several times to boost their nutrition^40^.

Our findings show how the displacement of native plants by IAPs that is happening across the Rift Valley and other parts of sub-Saharan Africa could increase *Culex* spp. larval density, adult populations, biting rate, survival and subsequently arboviral transmission. *Cx. pipiens* and several other *Culex* spp. are important vectors of West Nile virus, filariasis, encephalitic viruses and amplifiers of the Rift Valley fever virus during outbreaks^43^. The latter is of special concern in Baringo County which has been previously affected by outbreaks^44^ and where several other mosquito-borne arboviruses have been characterized. In Kenya (Baringo County), Tanzania and Ethiopia, cut stump and basal bark herbicide application methods are being utilized in the management of Prosopis^32^. On the other hand, management of Parthenium is based on traditional methods such as manual weeding, intensive tillage and herbicide application^9^. However, the use of suppressive legumes (*Lablab purpureus* and *Desmodium intortum*) have been successfully tested in Tanzania^5^. These strategies will also inadvertently mitigate the effects of IAPs on mosquito breeding and vectorial capacity.

One of the main limitations of the study is that our control treatment did not generate enough larvae for rearing in the insectary. While this was expected given that gravid mosquitoes are less likely to be attracted to sites that are not organically rewarding, it also meant that we did not have a baseline to compare body size and survival. Yet, we observed that once the control accumulated some soil derived organic matter the number of larvae increased. Therefore, in future studies, the controls could be artificially enriched by adding topsoil. In addition, several control containers could be set up to collect enough larvae for rearing. As this was a field-based experiment we had no control on the species composition and numbers emerging from our treatments. Despite these challenges our study provides valuable field-based insights on the influence of plant litter on habitats of container breeding *Culex* spp.

In conclusion, our study shows that IAP litter, especially that of Prosopis and Parthenium creates more favorable breeding conditions than native plants leading to enhanced *Culex* spp. larval abundance, adult mosquito size and longevity. Increased abundance and longevity of mosquitoes can greatly enhance their vectorial capacity for disease pathogens. Given the widespread encroachment of IAPs in Africa, this may contribute to increased mosquito populations with higher vectorial capacity and potentially enhance arbovirus transmission risks. Managing the spread of invasive vegetation could therefore serve as an important ecological intervention to curb mosquito breeding and arboviral disease burden. In future studies, a better understanding of the mechanisms mediating these IAP effects should be sought, including plant decomposition rates, phytochemicals, microbial composition and the chemical cues responsible for attraction of gravid mosquitoes.

## Electronic supplementary material

Below is the link to the electronic supplementary material.

Supplementary Material 1

## Data Availability

The dataset generated and analyzed in this study is available on the University of Liverpool Research Data Catalogue with the following DOI: https://doi.org/10.17638/datacat.liverpool.ac.uk/2966.
