# Supplementary material for: Invasive alien plant litter influences larval density, size and survival of Culex spp
Source: Sci Rep. 2025 Jul 24;15:26978. doi: 10.1038/s41598-025-11556-z (PMC12290050; doi:10.1038/s41598-025-11556-z)
Supplement: Supplementary file 1 — Supplementary Material 1 [file 41598_2025_11556_MOESM1_ESM.docx]

**Table**: Diversity of adult mosquitoes emerging from plant litter treatments in the two sampling periods

| **I** | | | | | | | | | | | | |
| --- | --- | --- | --- | --- | --- | --- | --- | --- | --- | --- | --- | --- |
|  | Acacia | | Lantana | | Parthenium | | Prosopis | | Control | | Total | |
| Species | **F** | **M** | **F** | **M** | **F** | **M** | **F** | **M** | **F** | **M** | **F** | **M** |
| *Culex pipiens* | 64 | 10 | 18 | 13 | 31 | 57 | 37 | 10 | - | - | 150 | 90 |
| *Cx. poicilipes* | - | - | - | - | - | - | 1 | - | - | - | 1 | - |
| *Cx. tigripes* | - | - | - | - | - | - | - | - | 1 | - | 1 | - |
| *Cx. vansomereni* | 4 | 15 | 7 | 9 | 40 | 50 | 12 | 17 | 29 | 17 | 92 | 108 |
| *Cx. watti* | 13 | - | 2 | - | 4 | - | 3 | - | - | - | 22 | - |
| *Cx. zombaensis* | 1 | - | 2 | 20 | 3 | 3 | 7 | 28 | - | - | 13 | 51 |
| **Subtotal** | 82 | 25 | 29 | 42 | 78 | 110 | 60 | 55 | 30 | 17 | 279 | 249 |
| **II** | | | | | | | | | | | | |
| *Culex annulirostris* | - | - | 7 | - | 2 | 1 | 19 | 14 | - | - | 28 | 15 |
| *Cx. cinereus* | - | - | 10 | 3 | - | - | 25 | 19 | - | - | 35 | 22 |
| *Cx. pipiens* | 106 | 26 | 114 | 43 | 127 | 63 | 47 | 35 | - | - | 394 | 167 |
| *Cx. poicilipes* | - | - | - | - | - | 1 | 1 | - | - | - | 1 | 1 |
| *Cx. tigripes* | 1 | - | 2 | 1 | - | 1 | - | 1 | - | - | 3 | 3 |
| *Cx. univittatus* | - | - | - | - | - | - | 2 | - | - | - | 2 | - |
| *Cx. vansomereni* | 3 | 8 | 7 | 58 | 42 | 18 | 4 | 11 | - | - | 56 | 95 |
| *Cx. zombaensis* | 29 | 125 | 85 | 83 | 34 | 55 | 5 | 9 | - | - | 153 | 272 |
| **Subtotal** | 139 | 159 | 225 | 188 | 205 | 139 | 103 | 89 |  |  | 672 | 575 |
